# Identification of Proteomic Biomarkers and Therapeutic Targets for Vitiligo Using a Two‐Sample Proteome‐Wide Mendelian Randomization Approach

**DOI:** 10.1111/jocd.70420

**Published:** 2025-08-26

**Authors:** Linli Liu, Lingli Deng, Xingyu Pan, Jin Chen, Chunshui Yu

**Affiliations:** ^1^ Department of Dermatology Suining Central Hospital Suining Sichuan China; ^2^ Department of Dermatology The First Affiliated Hospital of Chongqing Medical University Chongqing China

**Keywords:** molecular docking, proteome‐wide analysis, two‐sample Mendelian randomization analysis, vitiligo

## Abstract

**Background:**

Vitiligo is a chronic autoimmune disorder characterized by melanocyte loss and depigmented skin patches. Effective treatment options are limited, and therapeutic progress has been hindered by incomplete understanding of its precise pathogenic mechanisms. We aimed to identify candidate protein biomarkers and therapeutic targets for vitiligo by integrating large‐scale proteomics and genomic data using Mendelian randomization (MR).

**Methods:**

Using two‐sample MR analysis, we leveraged genome‐wide association study (GWAS) data for vitiligo (131 cases and 207 482 controls of European descent) and proteomic data comprising 4907 plasma proteins from the Decode cohort (35 559 participants). Causal relationships between genetically predicted plasma protein levels and vitiligo risk were evaluated through five complementary MR methods, along with enrichment analyses to explore their biological implications. Further validation was conducted via independent transcriptomic datasets, single‐cell RNA sequencing, and molecular docking analysis to identify potential therapeutic compounds.

**Results:**

We identified seven proteins (HEPHL1, PRDX1, DEFA1, CSGALNACT2, HERC4, NDC80, and SPHK2) causally associated with vitiligo risk. Notably, HERC4 and NDC80 exhibited robust expression in vitiligo lesions across validation datasets. Functional enrichment analysis implicated these proteins in oxidative stress regulation, immune modulation, and cellular signaling pathways. Molecular docking analyses further highlighted potential therapeutic agents, including zoledronic acid and gramine.

**Conclusions:**

Our integrative MR analysis identified novel protein biomarkers and promising therapeutic targets for vitiligo, particularly HERC4 and NDC80. These findings offer potential opportunities for improved diagnosis and the development of targeted therapies, advancing precision medicine approaches for vitiligo management.

## 1 Introduction

Vitiligo is a chronic autoimmune skin disorder characterized by melanocyte loss, resulting in depigmented patches that significantly impact patients' psychosocial well‐being [1, 2]. Current therapeutic options remain suboptimal, with frequent relapses and limited long‐term efficacy, underscoring an urgent clinical need for novel biomarkers and targeted therapies [3].

Genome‐wide association studies (GWAS) have identified numerous genetic susceptibility loci for vitiligo, illuminating potential immunological and genetic pathways implicated in its pathogenesis [4]. However, GWAS alone cannot reliably distinguish causative factors from mere associations, limiting direct translation into clinical practice. Mendelian randomization (MR), a powerful genetic epidemiological approach, overcomes these limitations by utilizing genetic variants as instrumental variables, thus enabling causal inference between exposure (e.g., plasma protein levels) and disease outcomes [5, 6, 7]. Recently, proteome‐wide Mendelian randomization studies have successfully identified therapeutic targets in various autoimmune and inflammatory diseases [8, 9, 10, 11], yet such analyses remain scarce in vitiligo.

In this study, we integrated large‐scale proteomics data and vitiligo GWAS summary statistics using a two‐sample MR approach to systematically identify proteins causally associated with vitiligo risk. Two of these proteins, HERC4 and NDC80, were identified as central regulators in key cellular processes, such as immune response regulation and cell cycle regulation. HERC4, an E3 ubiquitin ligase, plays a critical role in immune modulation and DNA damage repair [12], while NDC80, a part of the kinetochore complex, is involved in microtubule attachment and cell division [13]. However, the roles of these proteins in vitiligo pathogenesis remain underexplored, making them important candidates for further investigation. Subsequently, we identified candidate proteins' functional relevance through bioinformatics enrichment analyses, independent transcriptomic datasets, and single‐cell RNA sequencing (scRNA‐seq) from vitiligo lesions. We further conducted drug enrichment analysis and molecular docking simulations to explore actionable therapeutic opportunities. This comprehensive integrative approach aims to identify clinically relevant biomarkers and potential therapeutic targets, thereby facilitating translational advances in vitiligo management.

## 2 Methods

### 2.1 Study Design

**FIGURE 1:**
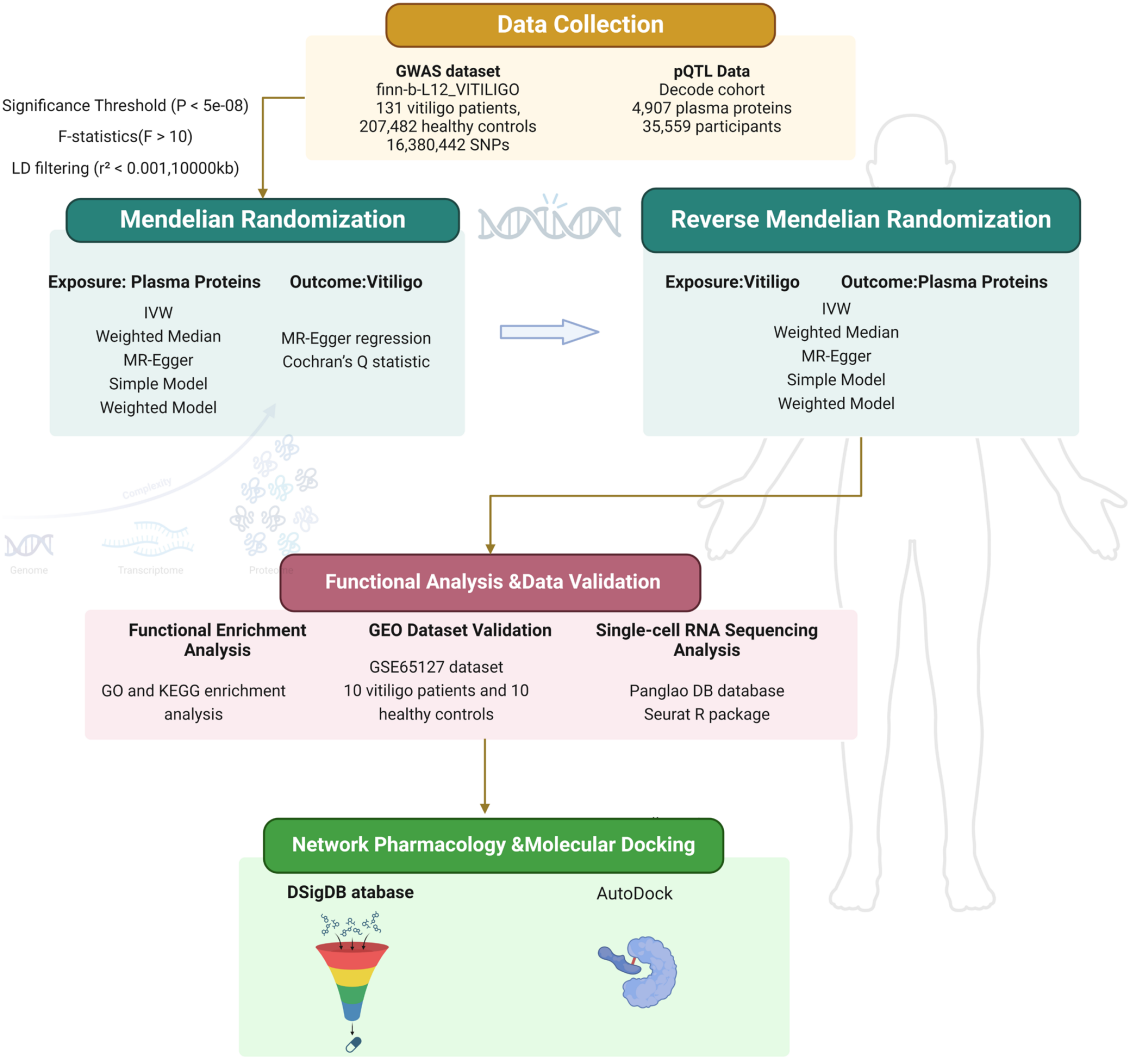
Study design and flowchart of proteome‐wide Mendelian randomization analysis for identifying potential therapeutic target genes in vitiligo. GEO, Gene Expression Omnibus; GWAS, genome‐wide association study; IVW, inverse variance weighting; MR, Mendelian randomization; PQTL, protein quantitative trait loci; SNP, single nucleotide polymorphism. The study incorporates two‐sample MR analysis to explore the causal relationship between the proteome‐wide dataset and vitiligo risk, integrating GWAS and PQTL datasets. Functional enrichment analyses, including Gene Ontology (GO) and KEGG pathway analysis, were performed for the candidate genes identified through two‐sample MR. Data validation was conducted using the GEO dataset (GSE65127) and single‐cell RNA sequencing, and molecular docking simulations were used to predict potential drug candidates for the identified target genes.

This study was performed following the guidelines of the STROBE‐MR checklist (https://www.strobe‐mr.org/). The overall study design comprised four main steps (Figure 1): (i) a discovery stage to identify plasma proteins causally associated with vitiligo risk using two‐sample proteome‐wide MR; (ii) sensitivity analyses and reverse MR to verify causal associations and evaluate pleiotropy; (iii) functional validation using transcriptomic and single‐cell RNA sequencing (scRNA‐seq) datasets; and (iv) drug enrichment and molecular docking analyses to identify candidate therapeutic compounds targeting the proteins identified in previous steps (Figure 1).

### 2.2 Data Sources

Genetic association data for vitiligo were sourced from the GWAS dataset (finn‐b‐L12_VITILIGO), which included 131 vitiligo cases and 207 482 controls of European descent, covering over 16 million SNPs. This imbalance in the number of vitiligo cases compared to controls may affect statistical power and false discovery rates. To mitigate this, stringent statistical thresholds were applied, and sensitivity analyses were performed to ensure the robustness of our findings. Proteomic data comprised cis protein quantitative trait loci (cis‐pQTL) for 4907 plasma proteins from 35 559 participants from the Decode cohort [14]. To validate identified proteins, we employed the publicly available transcriptomic GEO dataset (GSE65127) and single‐cell RNA sequencing data from PanglaoDB [15]. Detailed information on these datasets is summarized in Table S1.

### 2.3 Two‐Sample Mendelian Randomization Analysis

We applied a two‐sample MR analysis to evaluate causal relationships between genetically predicted plasma protein levels (exposures) and vitiligo risk (outcome). SNPs were selected as instrumental variables based on the stringent criteria: genome‐wide significance (*p <* 5 × 10^−8^), independence (linkage disequilibrium clumping at *r* ^2^ < 0.001 within 10 Mb), and instrument strength (*F*‐statistics > 10) [16]. After harmonization between exposure and outcome datasets, causal estimates were primarily obtained using inverse variance weighted (IVW) MR analysis [17]. Four additional MR methods—weighted median, MR‐Egger, simple mode, and weighted mode—were conducted for robustness checks [18]. Sensitivity analyses were performed using Cochran's *Q* test, MR‐Egger intercept tests, and MR‐PRESSO to identify heterogeneity and horizontal pleiotropy [19].

### 2.4 Reverse Mendelian Randomization Analysis

To confirm causality directionality, we conducted reverse MR analyses, assessing whether genetic liability to vitiligo affects plasma protein levels. MR estimates were obtained and assessed using similar approaches as described above, with robust sensitivity tests (Cochran's *Q*, MR‐PRESSO, MR‐Egger intercept) to address horizontal pleiotropy and potential bias.

### 2.5 Functional Enrichment and Drug Enrichment Analysis

Proteins causally linked to vitiligo were subjected to Gene Ontology (GO) and Kyoto Encyclopedia of Genes and Genomes (KEGG) enrichment analyses to elucidate biological functions and related pathways. Additionally, we conducted drug enrichment analysis using the DSigDB database, followed by molecular docking simulations to identify potential therapeutic candidates targeting causally implicated proteins. Molecular docking was performed with AutoDock Vina software, with 3D structures retrieved from PubChem and the Protein Data Bank.

## 3 Results

### 3.1 Proteome‐Wide MR Identified Seven Proteins Associated With Vitiligo Risk

**FIGURE 2:**
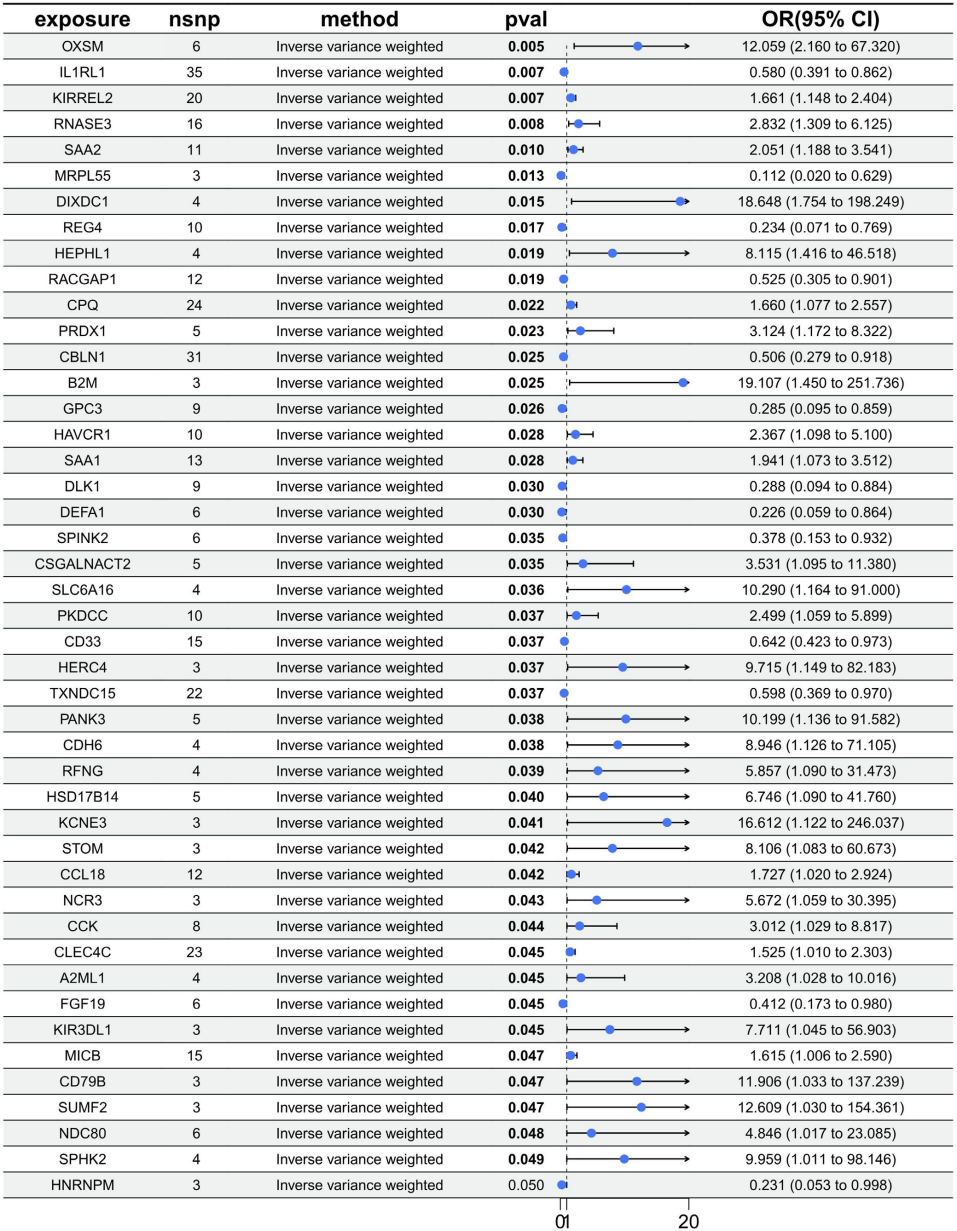
Forest plot of the MR estimates for the associations between 46 proteins and Vitiligo. The inverse variance weighted method is considered the main approach. CI, confidence interval; OR, odds ratio.

**FIGURE 3:**
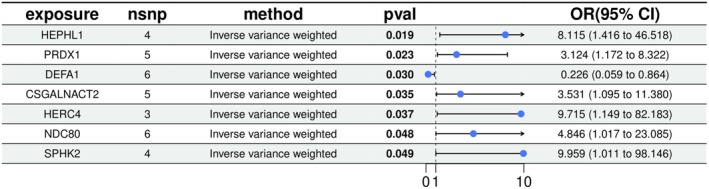
Forest plot of the reverse MR estimates. The inverse variance weighted method is considered the main approach. CI, confidence interval; OR, odds ratio.

**FIGURE 4:**
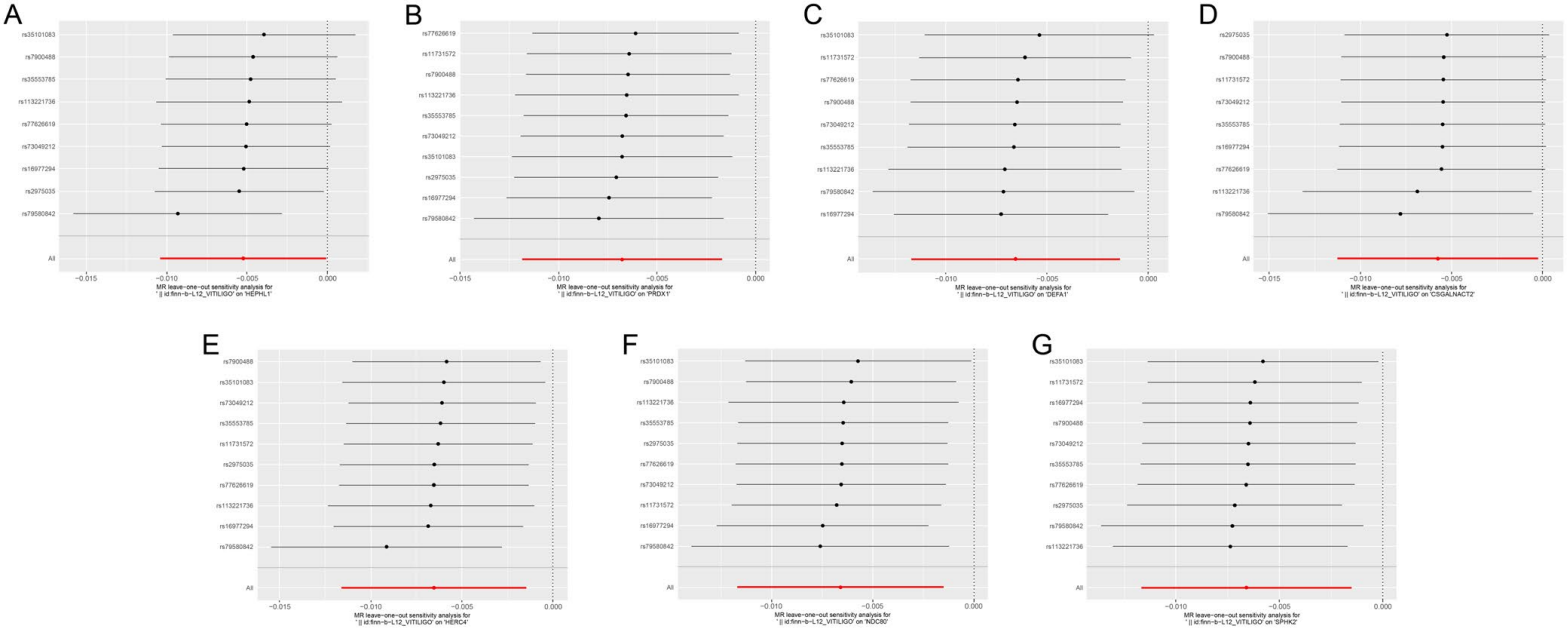
Scatter plots results of MR analysis between seven identified proteins and vitiligo. (A) HEPHL1, (B) PRDX1, (C) DEFA1, (D) CSGALNACT2, (E) HERC4, (F) NDC80, (G) SPHK2. SNP, single nucleotide polymorphism.

**FIGURE 5:**
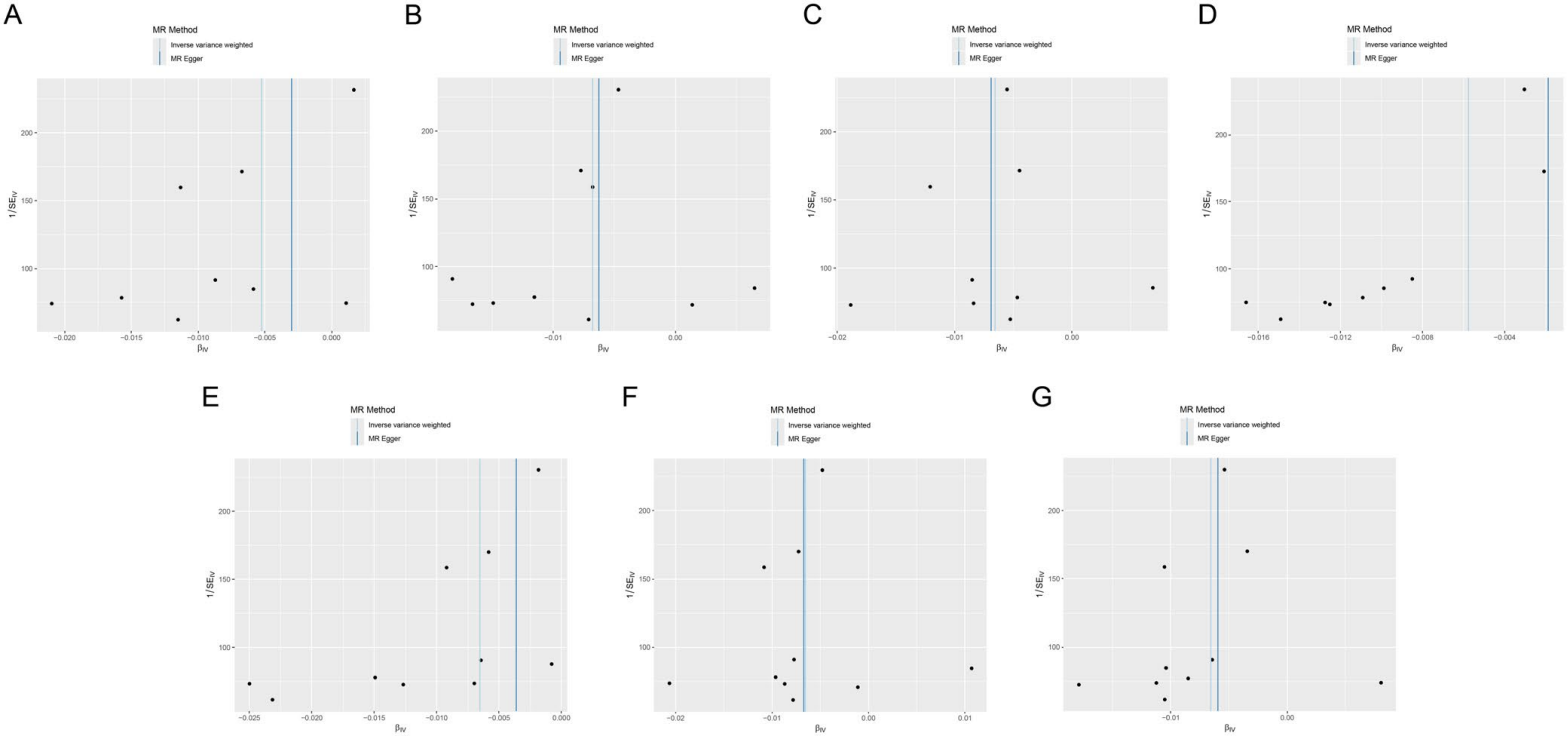
Leave‐one‐out sensitivity analysis results of MR analysis between seven identified proteins and Vitiligo. (A) HEPHL1, (B) PRDX1, (C) DEFA1, (D) CSGALNACT2, (E) HERC4, (F) NDC80, (G) SPHK2. SNP, single nucleotide polymorphism.

**FIGURE 6:**
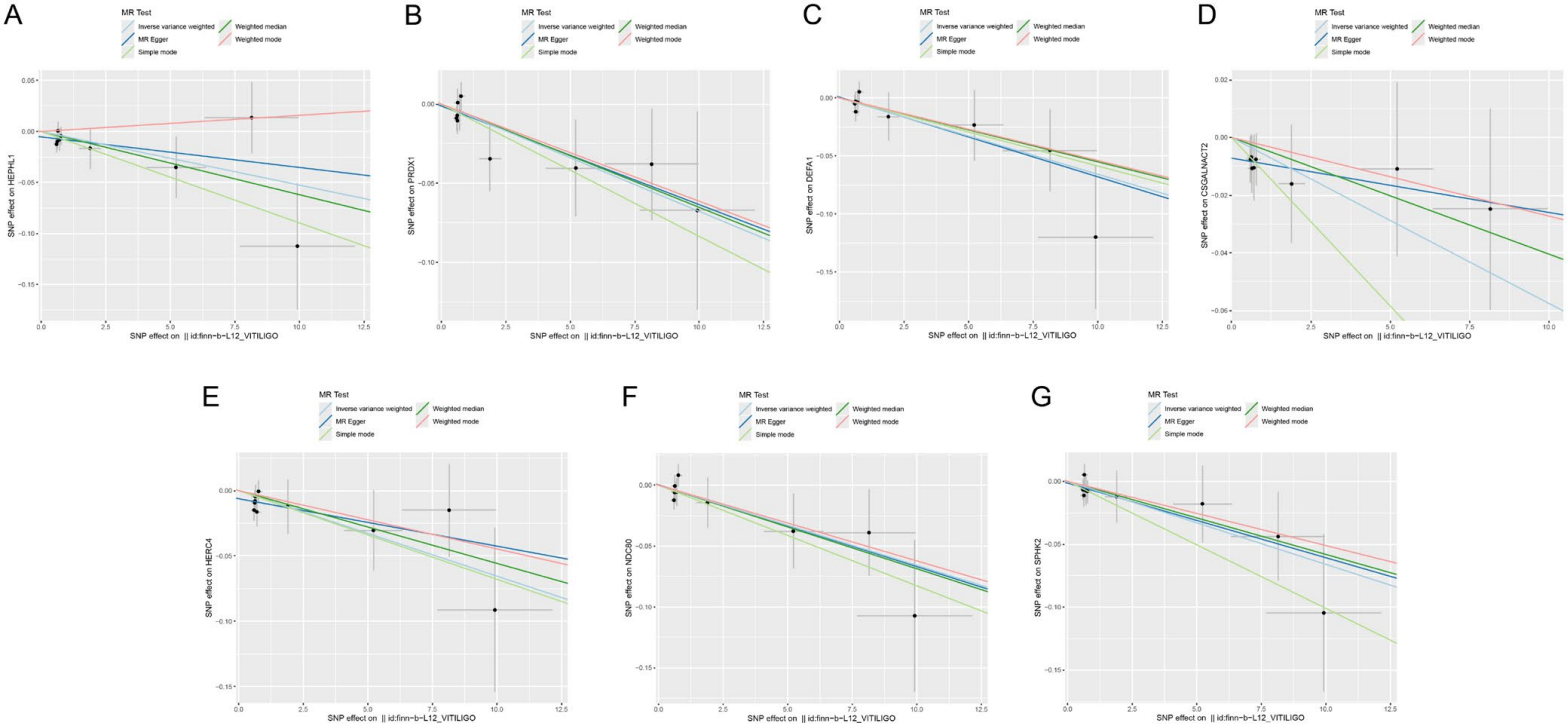
The funnel plots demonstrated the distribution of seven candidate proteins' instrumental variables. (A) HEPHL1, (B) PRDX1, (C) DEFA1, (D) CSGALNACT2, (E) HERC4, (F) NDC80, (G) SPHK2. SNP, single nucleotide polymorphism.

Based on the methodological framework established above, we next present the proteome‐wide MR findings. A proteome‐wide MR analysis identified 46 proteins significantly associated with vitiligo risk (*p* < 5e‐08) (Figure 2). These associations were based on robust instrumental variables selected following stringent LD filtering (*r* ^2^ < 0.001 within 10 000 kb) and weak genetic instruments (*F*‐statistic < 10). To further validate the robustness and causality of these findings, reverse MR analysis was performed, evaluating the potential causal influence of genetically predicted vitiligo on protein expression levels. Among these identified proteins, seven showed consistent and robust associations with vitiligo risk. These proteins include HEPHL1 (OR: 8.115, 95% CI: 1.42–46.52, *P*_IVW_ = 0.018), PRDX1 (OR: 3.124, 95% CI: 1.17–8.32, *P*_IVW_ = 0.023), DEFA1 (OR: 0.226, 95% CI: 0.06–0.86, *P*_IVW_ = 0.030), CSGALNACT2 (OR: 3.531, 95% CI: 1.10–11.38, *P*_IVW_ = 0.035), HERC4 (OR: 9.715, 95% CI: 1.15–82.18, *P*_IVW_ = 0.037), NDC80 (OR: 4.846, 95% CI: 1.02–23.09, *P*_IVW_ = 0.048), and SPHK2 (OR: 9.959, 95% CI: 1.01–98.15, *P*_IVW_ = 0.049) (Figure 3). Scatter plots demonstrating the MR estimates for these seven proteins are provided in Figure 4. Sensitivity analyses, including leave‐one‐out (LOO) analysis, demonstrated that individual SNPs did not significantly influence the robustness of these associations (Figure 5). Funnel plots further confirmed the validity of the MR assumptions, showing symmetrical distributions of SNPs without substantial bias (Figure 6).

Reverse MR analysis revealed no significant reverse associations for the seven identified proteins (*P*_IVW_ > 0.05 for all), indicating that genetically predicted vitiligo does not influence protein expression levels. Furthermore, the *p*‐values for reverse MR analysis were consistently greater than 0.05 for all seven proteins, suggesting no borderline significant reverse associations. These findings are further supported by Table 1, which demonstrates no evidence of pleiotropy or heterogeneity (*p* > 0.05 for all), reinforcing the validity of the causal direction observed in the forward MR analysis. Taken together, these results support the hypothesis that the identified proteins may contribute to vitiligo pathogenesis, with the reverse MR findings further validating the causal relationship between these proteins and vitiligo risk.

**TABLE 1:** Horizontal pleiotropy and heterogeneity analysis for the seven identified proteins.

Proteins	Pleiotropy		Heterogeneity			
	MR‐Egger		MR‐Egger		IVW	
	Intercept test					
	Intercept	*p*	*Q*‐statistic	*p*	*Q*‐statistic	*p*
HEPHL1	−0.00531	0.308	4.872	0.676	6.079	0.638
PRDX1	−0.00118	0.802	3.895	0.866	3.962	0.914
DEFA1	0.000685	0.891	3.147	0.871	3.167	0.923
CSGALNACT2	−0.00718	0.166	0.279	0.999	2.666	0.953
HERC4	−0.00621	0.205	3.219	0.919	5.126	0.823
NDC80	0.000296	0.949	4.067	0.851	4.071	0.907
SPHK2	−0.0014	0.757	2.799	0.946	2.901	0.968

### 3.2 Visualization and Functional Analysis of Seven Vitiligo‐Associated Proteins

**FIGURE 7:**
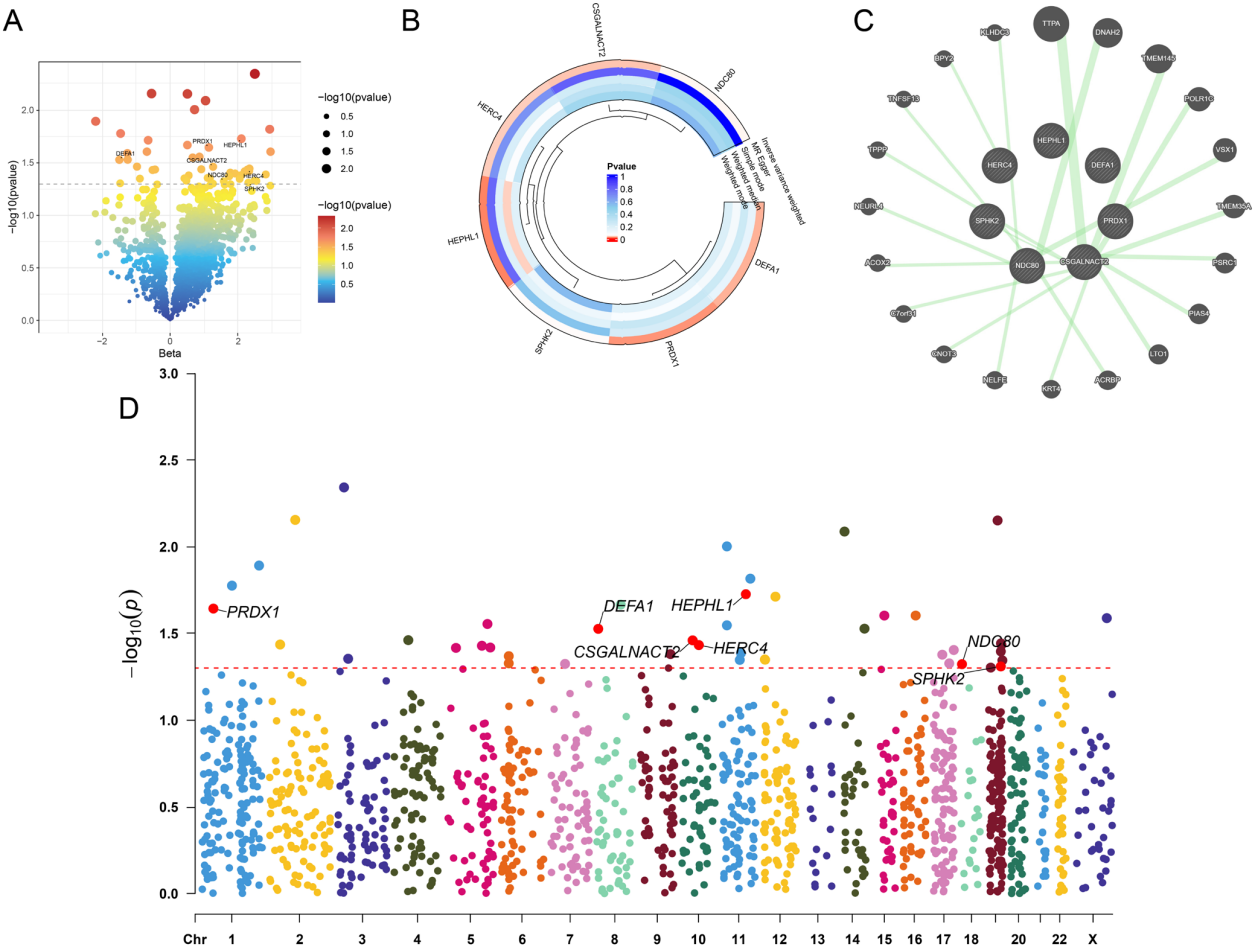
Illustration of the seven key genes identified through two‐sample Mendelian randomization analysis associated with vitiligo. (A) Volcano plot showing the significance and effect sizes of genetic variants for the candidate genes. (B) Circos plot visualizing the relationships and associations among the seven significant candidate genes and their instrumental variables. (C) Protein–protein interactive (PPI) network by the using GeneMANIA platform. (D) Manhattan plot highlighting the genomic distribution of genetic variants associated with the selected proteins and significant genes.

Genetic and functional characteristics of the seven vitiligo‐associated proteins were explored using multiple visualization methods, including volcano, circos, and Manhattan plots, as well as a protein–protein interaction (PPI) network analysis. The volcano plot effectively illustrated the significance and effect sizes of genetic variants linked to the identified proteins (Figure 7A). The circos plot clearly depicted associations between candidate proteins and their corresponding instrumental variables (Figure 7B), while the Manhattan plot showed the genomic distribution of these variants (Figure 7D). Additionally, the PPI network constructed using GeneMANIA highlighted interactions among the identified proteins and their related hub genes, providing further insight into their potential functional relationships in vitiligo pathogenesis (Figure 7C).

### 3.3 Functional Enrichment Analyses Suggest Involvement in Oxidative Stress and Immune Modulation

**FIGURE 8:**
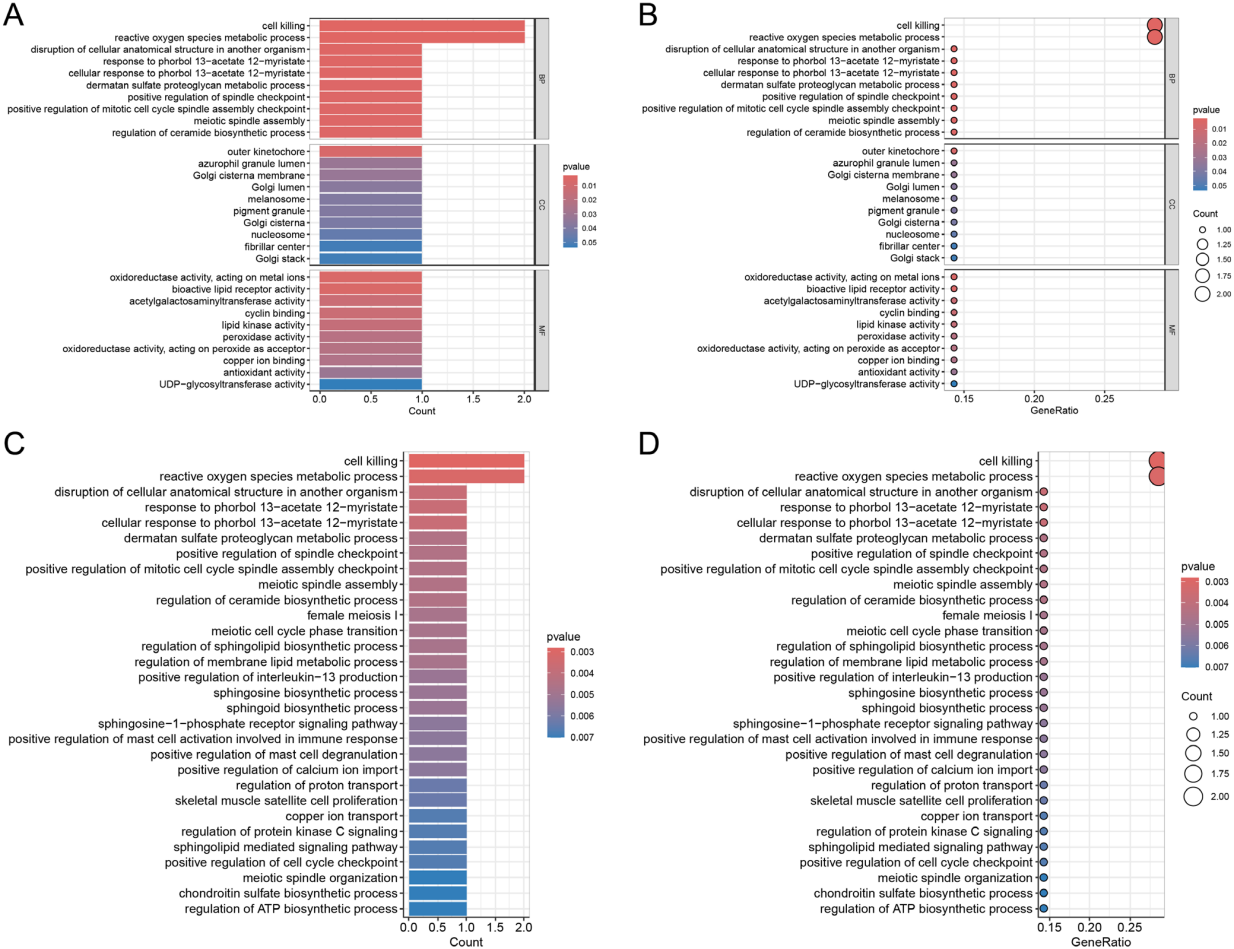
Functional enrichment analysis of seven identified genes. (A, B) Gene Ontology (GO) enrichment analysis of seven identified genes, including biological process (BP), cellular component (CC), and molecular function (MF) categories, highlighting key biological processes and molecular functions. Panel (A) barplot, panel (B) bubble plot. (C, D) KEGG pathway enrichment analysis. (C) Barplot, panel (D) bubble plot.

Functional roles of these proteins in vitiligo were further elucidated using GO enrichment and KEGG pathway analyses. GO analysis highlighted significant associations with biological processes (BP), including “cell killing,” “reactive oxygen species (ROS) metabolic process,” and “cellular response to phorbol esters,” suggesting their involvement in oxidative stress and immune‐mediated cytotoxicity—both pivotal in vitiligo pathogenesis. As illustrated in Figure 8A,B, cellular component (CC) enrichment revealed significant associations with “melanosome,” “pigment granule,” and “Golgi‐related compartments,” indicating potential roles of these proteins in melanocyte‐specific organelles and intracellular transport mechanisms. In molecular function (MF), significant associations were observed with “oxidoreductase activity,” “peroxidase activity,” and “antioxidant activity,” supporting the hypothesis that these proteins actively regulate redox homeostasis within melanocytes. As shown in Figure 8C,D, KEGG pathway analysis further identified critical pathways such as “ROS metabolic process,” “cell killing,” and “sphingolipid biosynthetic process,” which are closely related to vitiligo development.

### 3.4 Validation in Transcriptomic and Single‐Cell RNA Sequencing Data Confirmed Two Candidate Proteins

**FIGURE 9:**
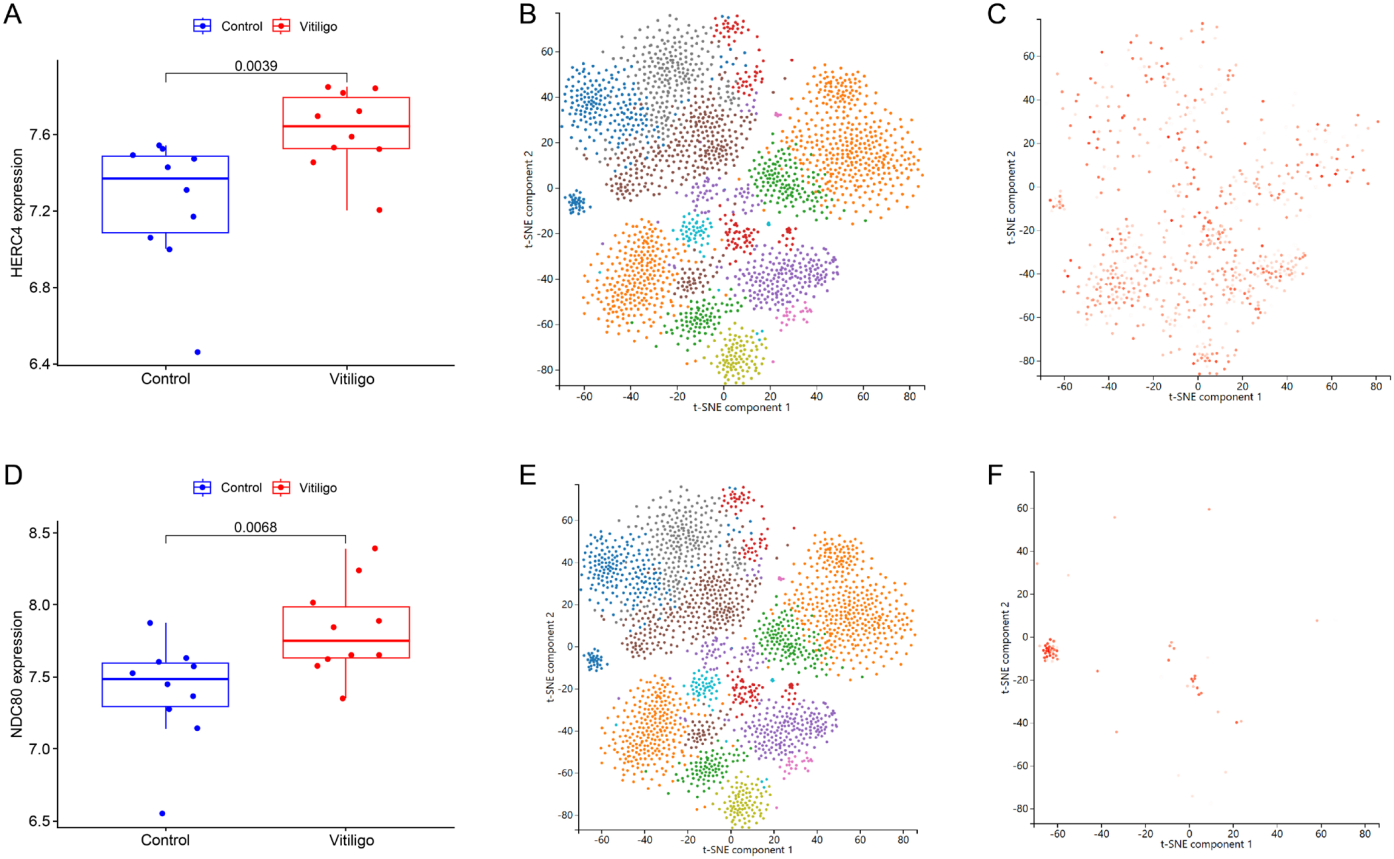
Validation of candidate genes in the GSE dataset and single‐cell RNA sequencing analysis. (A) Upregulation of HERC4 expression in the Vitiligo dataset (GSE65127, *p* = 0.0039). (B) Single‐cell RNA sequencing of skin tissue (SRA653146; SRS2874281) showing cellular distribution. (C) Enrichment of HERC4 expression in keratinocytes. (D) Upregulation of NDC80 expression in the Vitiligo dataset (GSE65127, *p* = 0.0068). (E) Single‐cell RNA sequencing of skin tissue (SRA653146:SRS2874281) showing cellular distribution. (F) Enrichment of NDC80 expression in keratinocytes.

We identified the elevated expression of HERC4 and NDC80 in vitiligo‐affected skin compared to healthy controls (*p* < 0.05) using the GEO dataset (GSE65127). We further confirmed the enrichment of these two proteins specifically in keratinocytes through single‐cell RNA sequencing (scRNA‐seq), indicating their potential mechanistic roles in vitiligo pathogenesis (Figure 9).

### 3.5 Identification of Potential Therapeutic Compounds via Molecular Docking Analyses

**FIGURE 10:**
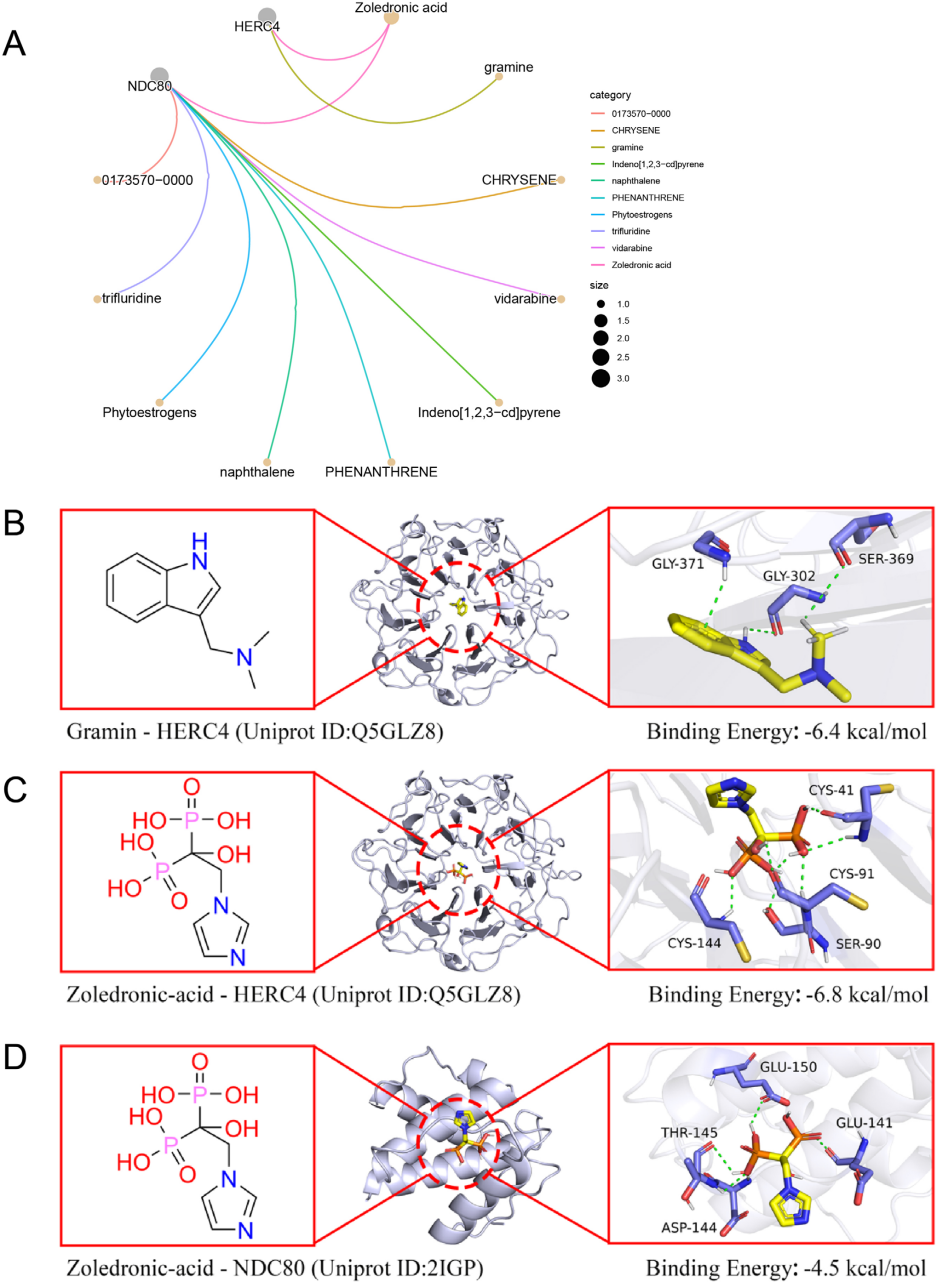
Drug enrichment and molecular docking analysis of NDC80 and HERC4. (A) Drug enrichment analysis of NDC80 and HERC4, identifying potential drug candidates. (B) Molecular docking of Gramine with HERC4. (C) Molecular docking of Zoledronic acid with HERC4. (D) Molecular docking of Zoledronic acid with NDC80.

Drug enrichment and molecular docking analyses identified zoledronic acid and Gramine as promising therapeutic candidates targeting HERC4 and NDC80. Both compounds exhibited strong binding affinity to target proteins, suggesting therapeutic potential pending experimental validation (Figure 10).

### 3.6 Summary of Findings

This integrative proteome‐wide MR approach identified seven plasma proteins causally associated with vitiligo. Among these, HERC4 and NDC80 emerged as robust candidate biomarkers, supported by functional enrichment, independent transcriptomic validation, and targeted molecular docking analysis for therapeutic exploration. These findings provide valuable insights into vitiligo's underlying pathogenesis and potential novel avenues for therapeutic intervention.

## 4 Discussion

In this study, we systematically identified seven proteins causally associated with vitiligo risk using a comprehensive two‐sample proteome‐wide MR approach. These proteins include HEPHL1, PRDX1, DEFA1, CSGALNACT2, HERC4, NDC80, and SPHK2. They are implicated in oxidative stress, immune regulation, and melanocyte dysfunction. These processes are central to vitiligo pathogenesis. Among these, HERC4 and NDC80 were robustly identified through independent transcriptomic and scRNA‐seq analyses, highlighting their potential as biomarkers or therapeutic targets. To our knowledge, this study is the first proteome‐wide MR analysis specifically addressing vitiligo, providing novel insights into its underlying mechanisms and potential drug targets.

Previous GWAS have identified over 50 genetic susceptibility loci for vitiligo, underscoring its immune‐related genetic foundation and relevance across populations [2]. However, GWAS findings alone do not establish causality due to potential confounding from linkage disequilibrium or environmental factors [20, 21]. Mendelian randomization, leveraging genetic variations linked to modifiable exposures, provides a robust method to infer causality and reduce confounding [22]. Indeed, MR has successfully identified causal biomarkers and therapeutic targets in autoimmune diseases, including rheumatoid arthritis, lupus, and Sjögren's syndrome [23, 24, 25]. Our study integrates GWAS with proteomic quantitative trait loci (PQTL) data, a strategy previously effective in uncovering causal proteins in other complex diseases but not yet applied to vitiligo [26, 27]. This novel application provides fresh insights into disease pathogenesis and potential therapeutic targets.

Our proteome‐wide MR analysis identified seven proteins significantly associated with vitiligo, implicated primarily in oxidative stress, immune regulation, and cellular signaling. Oxidative stress, a hallmark of melanocyte dysfunction in vitiligo, contributes directly to melanocyte death [28]. Immune‐mediated cytotoxicity, particularly involving CD8+ T cells, further exacerbates melanocyte destruction [29, 30]. Among the identified proteins, SPHK2 plays a pivotal role in sphingolipid metabolism, specifically in the synthesis of sphingosine‐1‐phosphate (S1P), which regulates both oxidative stress and immune responses. S1P, synthesized by SPHK2, regulates immune cell trafficking, inflammation, and oxidative stress, all of which are central to autoimmune diseases like vitiligo [31, 32]. Dysregulation of SPHK2 may exacerbate these inflammatory processes, contributing to melanocyte destruction in vitiligo. Furthermore, SPHK2 has been shown to regulate mitochondrial function and calcium homeostasis, both of which are crucial for cellular stress responses and survival [33, 34].

In addition, analysis of the GEO dataset (GSE65127) identified the significant upregulation of HERC4 and NDC80 in vitiligo lesions. HERC4, an E3 ubiquitin ligase involved in DNA damage repair, cellular growth, and immune regulation [12], has previously been associated with cancers including breast and lung cancer [35, 36]. NDC80, involved in mitotic spindle assembly, has been recognized as a biomarker in various autoimmune conditions, including psoriasis and type 1 diabetes [37, 38, 39, 40]. Our scRNA‐seq validation further confirmed that both HERC4 and NDC80 are predominantly expressed in keratinocytes. Keratinocytes contribute significantly to vitiligo pathogenesis through secretion of chemokines such as CXCL9 and CXCL10, sustaining immune cell recruitment and inflammation [41]. Furthermore, keratinocyte‐specific oxidative stress, evidenced by reduced AQP3 expression, directly induces melanocyte apoptosis in vitiligo [42]. Recent studies have shown that NDC80 regulates oxidative stress, modulates immune responses, and influences autophagy and ROS production, all of which may affect melanocyte survival in vitiligo [43, 44, 45]. Similarly, HERC4 regulates immune signaling, cell growth, and protein degradation, which may impact keratinocyte function and contribute to immune dysregulation in vitiligo [12]. Taken together, these findings suggest that both HERC4 and NDC80, through their roles in immune modulation, oxidative stress, and cell survival, likely contribute to vitiligo pathogenesis by altering keratinocyte‐melanocyte interactions.

Moreover, drug enrichment and molecular docking analyses highlighted Zoledronic acid and Gramine as promising therapeutic agents targeting HERC4 and NDC80. Zoledronic acid, previously shown to inhibit the JAK/STAT3 Pathway—a crucial axis in autoimmune diseases [46, 47]‐may modulate immune responses relevant to vitiligo. Gramine, an antioxidant known to suppress NF‐κB and JAK/STAT3 signaling [48], offers dual potential in alleviating oxidative stress and immune dysregulation associated with vitiligo. However, it is important to emphasize that in silico docking results do not confirm biological efficacy or safety. These are preliminary findings that warrant further validation in preclinical models to confirm their therapeutic efficacy. This study has several methodological strengths. First, the two‐sample MR approach mitigates biases inherent to observational studies, enhancing causal inference robustness. Second, comprehensive sensitivity analyses, including reverse MR, increased the reliability of identified protein associations. Third, the integrative analysis spanning proteomics, transcriptomics, and molecular docking represents a novel and powerful strategy for biomarker discovery and therapeutic target prioritization in vitiligo research.

However, our study also has limitations. First, the MR analyses relied solely on genetic summary statistics from European populations, limiting generalizability across different ancestries. Second, the sample size of the validation GEO dataset (10 vitiligo cases) was small, potentially reducing the statistical robustness of findings. Third, scRNA‐seq validation confirms only mRNA‐level expression; thus, protein‐level confirmation is required given potential discrepancies due to post‐translational regulation. Finally, molecular docking provides preliminary therapeutic candidates; however, biological validation through in vitro or in vivo models remains necessary.

Future studies should address these limitations by incorporating larger, multi‐ethnic cohorts, longitudinal analyses of protein expression, and functional assessments in cellular or animal models. Given that Zoledronic acid and Gramine emerged as promising therapeutic compounds, further experimental investigation is essential to verify their therapeutic efficacy and safety profiles. In‐depth mechanistic studies of HERC4 and NDC80 in melanocyte biology and immune interactions would also provide insights necessary to advance these targets toward clinical application.

In summary, our integrative proteome‐wide MR analysis identified seven proteins causally associated with vitiligo risk. Among them, HERC4 and NDC80 were identified as promising biomarkers and potential therapeutic targets. Further functional validation and preclinical investigations will facilitate the translation of these findings into improved clinical diagnosis and targeted therapeutic strategies, offering significant promise for precision medicine approaches in vitiligo management.

## Author Contributions

Linli Liu, Jin Chen, and Chunshui Yu conceptualized and designed the study. Linli Liu performed the genetic and bioinformatic analyses and was responsible for data curation and formal analysis. Lingli Deng and Xingyu Pan contributed to data visualization by generating the figures. All authors participated in data interpretation and manuscript preparation. Linli Liu drafted the original manuscript, and all authors contributed to reviewing and editing the final version. All authors have accessed and verified the underlying data and approved the final manuscript.

## Ethics Statement

This study utilized publicly available summary‐level data from genome‐wide association studies (GWAS), proteomic datasets, and transcriptomic datasets (GSE65127).

## Consent

Ethical approval for these datasets was obtained in the original studies, and all participants provided informed consent. No new individual‐level data or human participants were involved in this study, and therefore, additional ethical approval was not required.

## Conflicts of Interest

The authors declare no conflicts of interest.

## Supporting information


**Data S1:** jocd70420‐sup‐0001‐TableS1.docx.

## Data Availability

The data that support the findings of this study are available from the corresponding author upon reasonable request.
