# Supplementary material for: Identification of Proteomic Biomarkers and Therapeutic Targets for Vitiligo Using a Two‐Sample Proteome‐Wide Mendelian Randomization Approach
Source: J Cosmet Dermatol. 2025 Aug 26;24(9):e70420. doi: 10.1111/jocd.70420 (PMC12379178; doi:10.1111/jocd.70420)
Supplement: Supplementary file 1 — Data S1: jocd70420‐sup‐0001‐TableS1.docx. [file JOCD-24-e70420-s001.docx]

**Table S1. Brief characteristics description of vitiligo genetic association data, proteomic data, and validation datasets used in this study.**

| **Exposure or outcome** | **Source** | **Sample size** | **Ancestry** | **Access Link** | **PMID** |
| --- | --- | --- | --- | --- | --- |
| Vitiligo Genetic Association Data | finn-b-L12_VITILIGO GWAS | 131 cases and 207,482 controls | European | <https://gwas.mrcieu.ac.uk/datasets/> | / |
| Proteomic Data | Decode cohort | 4,907 plasma proteins from 35,559 participants | European | <https://www.decode.com/summarydata/> | / |
| GEO Dataset  (GSE65127) | GEO | 10 vitiligo patients and 10 healthy controls | European | <https://www.ncbi.nlm.nih.gov/geo/> | / |
| Single-Cell RNA Data | PanglaoDB | Not specified | European | <https://panglaodb.se/> | / |
